# Post-awakening cortisol and resilience: unravelling their impact on cognitive decline

**DOI:** 10.1007/s00702-025-02958-4

**Published:** 2025-06-12

**Authors:** Pablo Rivas-Diaz, David Trillo-Figueroa, Valerie Rodriguez-Hernandez, Noemi SanMiguel, Vanesa Hidalgo, Alicia Salvador

**Affiliations:** 1 Laboratory of Social and Cognitive Neurosciences, Department of Psychobiology, University of Valencia, Valencia, Spain; 2 Department of Psychology and Sociology, Area of Psychobiology, University of Zaragoza, Campus Ciudad Escolar, Teruel, 44003 Spain; 3 Spanish National Network for Research in Mental Health CIBERSAM, Madrid, 28029 Spain

**Keywords:** Post-awakening cortisol, Verbal fluency, Executive function, Declarative memory, Resilience

## Abstract

Age-associated cognitive decline is a growing social problem and a major concern for older adults, highlighting the need to identify modifiable factors to mitigate it. The basal functioning of the hypothalamic-pituitary-adrenal (HPA) axis, and, especially, the post-awakening cortisol levels, seems to play a key role in cognitive performance related to functions dependent on the prefrontal cortex and hippocampus. Regulating these cortisol levels is crucial in this context, and resilience has been recognized as vital for successful aging and cognitive functioning. This study was designed to examine how post-awakening cortisol levels (both the cortisol awakening response, CAR (AUCi) and total post-awakening cortisol secretion, AUCg) could predict cognitive decline across different domains, and explore the role of resilience in this relationship. To investigate this, a follow-up study conducted between 2018 (Wave 1, W1) and 2022 (Wave 2, W2) included 53 healthy adults who completed tasks assessing verbal fluency, executive function, declarative memory, and resilience in both waves to study the cognitive decline. Moreover, in W1, participants provided four morning saliva samples on two consecutive working days to assess post-awakening cortisol levels on days that were different from the cognitive testing sessions. Results showed a negative association between AUCg and both phonemic and semantic fluency decline, but not in other cognitive domains, indicating a beneficial effect of post-awakening cortisol secretion on cognitive maintenance that appears be domain specific. In addition, resilience positively mediated the association between the CAR and the maintenance of semantic fluency. These findings underscore the role of post-awakening cortisol levels in supporting resilience and protecting prefrontal cortex-dependent functions, such as semantic fluency, over functions reliant on the hippocampus.

## Introduction

Decline in cognitive functioning is a pressing concern for a significant portion of the population, with studies indicating that 30–60% of older adults express apprehensions about this issue (Werner 2004; Cantegreil-Kallen and Pin 2012). This concern is aggravated by the rapid aging of the global population. According to the United Nations, by 2050, one in six people worldwide will be 65 years old or more, compared to one in eleven in 2019. This situation underscores the critical need to identify modifiable factors that can mitigate cognitive decline in older age. Although cognitive deterioration is influenced by several general factors, the stress response, and particularly the basal functioning of the hypothalamic-pituitary-adrenal (HPA) axis and the related cortisol changes, emerges as a pivotal element.

Cortisol secretions adhere to a circadian rhythm, typically initiating a gradual increase in the second part of the night when levels are at their lowest (O’Hara et al. 2007), peaking 30–45 min post-awakening, and reaching lower levels at the end of the day. Regarding post-awakening cortisol levels, according to Stalder et al. (2024), two indices can be obtained: (i) the change in levels with reference to the awakening level and (ii) the absolute levels during the post-awakening period. The former can be known as the cortisol awakening response (CAR), and it is usually calculated considering the Area Under the Curve with respect to the increase (the CAR itself or AUCi). The latter represents the total post-awakening cortisol secretion, and it is calculated as the Area Under the Curve with respect to the ground (AUCg). Both indices are widely regarded as representative measures of post-awakening cortisol levels, and they are assumed to prepare the body for the cognitive, physical, and emotional demands of the day by supplying the essential energy needed for the shift from a state of rest to one of activity (Stalder et al. 2024). Although these two indices are mathematically related, AUCg is less responsive to short-term fluctuations than CAR, and it is considered more stable over time (Stalder et al. 2016), which may lead to distinct effects on cognition and emotion regulation.

A systematic review by Law and Clow (2020) explored the relationship of the post-awakening cortisol response with different cognitive domains, proposing that it could act as a time-of-day marker. This proposal could potentially explain associations between the magnitude of the CAR and the function of brain regions with a high affinity for cortisol, such as the prefrontal cortex and hippocampus, which are mainly associated with executive functions and declarative memory, respectively. Regarding the prefrontal cortex, and according to the idea presented in Mizoguchi et al. (2004) that endogenous glucocorticoids in this area modulate cognitive functions, existing literature, such as Dierolf et al. (2016), generally shows a positive association between cortisol levels and prefrontal cortex functions. One of the most well-known prefrontal cortex-dependent functions is executive functioning, which Diamond (2013) divided into three main subcategories: inhibition, working memory, and cognitive flexibility. Thus, Geerlings et al. (2015) found a positive relationship between higher CAR and the inhibition capacity, assessed using the Stroop III test. In the case of working memory, Moriarty et al. (2014) observed a positive association with morning cortisol levels, specifically post-awakening cortisol secretion (AUCg one hour after awakening), rather than the CAR itself (AUCi). Additionally, cognitive flexibility or set shifting has been identified as an executive function more consistently linked to the CAR (Evans et al. 2012). However, Dierolf et al. (2016) suggested that this association is more closely tied to acute cortisol administration depending on basal cortisol levels. These basal levels were assessed using the AUCg within the first hour after awakening, a measure more closely related to total post-awakening cortisol secretion than to the CAR itself.

Regarding hippocampus-dependent memory, Law and Clow 2020 proposed that the CAR may negatively affect declarative memory, as previously found by Almela et al. (2012) and Hidalgo et al. (2016), although some studies presented a positive association (Ennis et al. 2016) or even no relationship between the CAR and declarative memory (Evans et al. 2012). The majority of the published studies employed cross-sectional rather than longitudinal designs (Ouanes et al. 2020; Tsui et al. 2020), yielding contradictory findings. Thus, Tsui et al. (2020) failed to identify a prospective effect of the CAR on cognitive change over a 5-year follow-up period, whereas Ouanes et al. (2020) reported slowness in the increase or even a decrease in the CAR peak, calculated as the cortisol level measured 30 min after awakening, as cognitive decline progresses. This gap in the literature in terms of longitudinal research is particularly relevant, given that the CAR may be a sensitive predictor of long-term cognitive changes. Recent evidence highlights the role of the CAR in modulating synaptic plasticity and cognitive functions (Kalafatakis et al. 2018; Liston et al. 2013). Cortisol effects accumulate over time, which supports the idea that chronic stress gradually disrupts neurocognitive processes, ultimately impairing cognition (Law and Clow 2020).

Given the well-established link between cortisol and cognitive performance, managing cortisol levels may play an intermediary role in this relationship. As previously mentioned, post-awakening cortisol levels prepare the body not only for cognitive, but also for emotional challenges (Stalder et al., 2024). Specifically, the CAR may attenuate the stress-distress association (Powell and Schlotz 2012) and counter-regulate prior-day emotional experiences, which can be exacerbated by increased prior-evening worrying and rumination (Zoccola et al. 2011), elevated stress levels (Kramer et al. 2019), and high chronic work overload (Schlotz et al., 2004). In this context, the ability to effectively regulate stress and emotions becomes crucial in maintaining cognitive function. Resilience, a concept closely tied to HPA axis regulation, plays a key role in this process, enabling individuals to sustain cognitive performance despite the challenges posed by aging or pathology (Yang et al. 2021). Indeed, resilience is a crucial component of successful aging (Musich et al. 2022) and overall well-being, and strengthening it has been recognized as a public health priority by the World Health Organization WHO (2022). Resilience impacts cognitive domains differently. More resilient individuals tend to score higher on working memory, verbal fluency, and processing speed (Fazeli et al. 2019), which are considered highly prefrontal cortex-dependent domains (Bonetti et al. 2019). This situates the prefrontal cortex as a key area for resilience (Eaton et al. 2022). However, this positive effect of resilience does not seem to occur on functions that are hippocampus-dependent, such as delayed recall (Fazeli et al. 2019). Overall, in terms of cognitive change, resilient individuals would be less susceptible to cognitive decline (Boyle et al. 2024). However, few studies have been carried out on resilience and cognitive functioning, which means that further longitudinal research is needed to better understand these dynamics (Saez-Sanz et al. 2023).

With this context in mind, the primary objective of this study was to investigate the impact of post-awakening cortisol levels (i.e., CAR and total post-awakening cortisol secretion or AUCg) on decline, over a period of four years, on cognitive domains dependent on hippocampal and prefrontal cortex functioning in healthy individuals. Given that post-awakening cortisol levels (Kupper et al. 2005) and the CAR (Wüst et al. 2000) show substantial heritability (32–40%), and considering the role of the CAR in modulating synaptic plasticity and its accumulating neurobiological effects (Stalder et al. 2024), we hypothesize that its impact on cognition may become evident when examining changes in cognitive performance four years later. Thus, we hypothesized that post-awakening cortisol, measured at baseline (Wave 1, W1), could either protect against or contribute to cognitive decline over time (Wave 2, W2), depending on the cognitive domain.

Based on this, we expected, on the one hand, that higher cortisol levels after awakening, particularly AUCg given its greater stability (Stalder et al. 2016), measured at W1, would be more strongly associated with protection against decline in prefrontal-dependent functions when assessed four years later (i.e., greater total post-awakening cortisol secretion, less decline in cognitive flexibility and executive functions) (Evans et al. 2012; Bäumler et al. 2014; Moriarty et al. 2014; Geerlings et al. 2015; Dierolf et al. 2016). On the other hand, and following previous literature, we expected a negative effect of baseline post-awakening cortisol levels on cognitive decline in hippocampal-dependent domains over the following four years (i.e. higher post-awakening cortisol levels, greater decline in declarative memory) (Almela et al. 2012; Hidalgo et al. 2016). Additionally, due to the close link between post-awakening cortisol, stress regulation, and cognitive performance or decline, we aimed to investigate the mediational role of resilience in this relationship. Given its role in stress adaptation, emotional regulation, and preserved cognitive functioning (Wolf et al. 2019; Yang et al. 2021), we propose that resilience would be a potential intermediary. Thus, we hypothesized that resilience would positively mediate the association between post-awakening cortisol and cognitive decline, particularly in cognitive domains dependent on prefrontal cortex functioning.

## Materials and methods

### Participants

All the participants were undergraduates from the University of Valencia (Spain) enrolled in a study program for people over 50 years of age called “Nau Gran”. They were native Spanish speakers and residents of the province where the study was conducted. In Wave 1 (W1, 2018–2019), 164 healthy adults (82 men and 82 women) (M = 43.36, SD = 21.69) participated in the study. The exclusion criteria at W1 were: (i) smoking more than 10 cigarettes a day, (ii) alcohol or other drug abuse, (iii) visual or hearing problems, (iv) diabetes, neurological, or psychiatric disease, (v) using any medication directly related to emotional or cognitive functioning or able to influence hormonal levels, such as glucocorticoids, psychotropic substances, or sleep medications, (vi) having been under general anesthesia once or more than once in the past year, and (vii) the presence of a stressful life event (e.g. death of a relative, divorce or separation, having been fired, serious personal illness, serious personal accident or injury, or serious illness in the family) in the past year. None of the participants scored less than 27 on the Spanish version of the Mini-Mental State Examination (MMSE) (Lobo et al. 1999), indicating the absence of cognitive impairment.

Four years later, in Wave 2 (W2, 2022–2023), participants were contacted by telephone and invited to take part in a follow-up study. Fifty-three individuals (24 men and 29 women) (M = 57.55, SD = 19.77) agreed to participate in this second wave (W2). All the participants met the same health criteria as in W1, except for three participants who scored less than 27 on the MMSE. Therefore, we carried out all the analyses with and without these three participants.

One hundred and eleven participants from W1 (58 men and 53 women) did not agree to participate in W2, which meant there was a high experimental attrition (67.68%). They declined our invitation for several reasons: (i) 17 said they were very busy and would not have time, (ii) nine refused without giving any reason, (iii) six had moved far away, (iv) one had passed away, and, finally, (v) 78 did not answer the phone or the phone numbers were erroneous.

This study was approved by the Ethics Committee of the University of Valencia (Code: 1034878) in accordance with the ethical standards of the Declaration of Helsinki. Participants signed the informed consent after receiving an explanation of the general procedure in the two waves.

### Procedure and cognitive assessment

In W1 and W2, participants were asked to attend a neuropsychological session at the Social Cognitive Neuroscience Laboratory (University of Valencia, Spain). Before each session, participants were interviewed by phone to obtain information about their general habits (e.g., alcohol consumption, smoking), including information about their medication, among other things, in order to find out whether they met the inclusion criteria (see Participants section). Moreover, participants were asked to maintain their general habits, that is, sleep as much as usual, refrain from heavy physical activity the day before the session, and not consume alcohol from the night before the session.

The cognitive assessment was conducted between 10.00 h and 12.00 h in both waves. In each session, participants completed several tests assessing different cognitive domains: verbal fluency, executive function, and declarative memory.

*Verbal fluency* To assess verbal fluency performance two distinct tests measuring phonemic and semantic fluency were used. To assess phonemic fluency, participants were told to generate as many words as possible starting with the letters F, A, and S. To evaluate semantic fluency, participants were asked to generate as many words as possible within the animal category. Each category had a time limit of 60 s. Only correct responses were scored, with intrusions, repeated attempts, and variations within the same species not being counted. Instructions were provided according to the administration procedures outlined in the Barcelona Test (Peña-Casanova 1991).

*Executive function* Two tasks were used to measure executive function, the Trail-Making Test (TMT; Reitan and Wolfson 1995) and the Stroop Color-Word Interference Test (Golden et al. 1978). The TMT consists of 25 circles distributed on a white sheet of paper. Participants were asked to trace a line connecting the circles as quickly as possible. The test consisted of two different forms: (i) The TMT-A consisted of circles containing 25 numbers that participants had to connect in numerical sequence. The TMT-A was used to assess general psychomotor speed and attention; (ii) The TMT-B included circles containing numbers from 1 to 25 and letters from A to L. Participants were instructed to connect numbers and letters in ascending order, alternating between numbers and letters. The TMT-B assessed the efficiency of their attention-switching performance. The outcome on each form was the time (in seconds) needed to complete the test, with less time indicating better performance. Golden’s version of the Stroop Color-Word Interference Test (Golden et al. 1978) comprises three distinct trials. The first trial features 100 color words printed in black ink (words trial; W); the second trial displays 100 “Xs” printed in different colors (red, green, and blue) (colors trial; C); and the third trial shows 100 color words from the first trial printed in colors that do not match the words (words-colors; WC). Participants were instructed to read the words on the first trial and then name the ink colors on the second and third trials as quickly and accurately as possible within a 45-second timeframe. The interference index, calculated according to the following formula: WC – (W×C)/(W + C), served as a measure of the ability to inhibit automatic responses, with higher scores reflecting better performance.

Declarative memory The Spanish version of the Free and Cued Selective Reminding Test (FCSRT) (Peña-Casanova et al. 2009) was used to measure declarative memory performance. This test consists of a list of 16 words, each belonging to a different semantic category, where the subject has to identify each word when answering a question (e.g., which one is a bird? ). Then, the distracting task starts, where the participant has to subtract numbers by 3s for 20 s. Afterwards, the free recall begins and lasts for 90 s. On the facilitated recall task, the experimenter asks participants facilitating questions about words they did not remember in the free recall part. The same process is repeated in three trials. The index used was the Total Immediate Recall (TIR), which represents the sum of the recall across three trials, including facilitated recall, with a maximum score of 48 points.

In addition, participants completed the short version (Campbell-Sills and Stein 2007) of the Spanish version (Notario-Pacheco et al. 2011) of the Connor-Davidson Resilience Scale (CD-RISC) to assess resilience. This 10-item scale, rated on a 5-point Likert scale (0 = “rarely” to 4 = “almost always”), evaluates an individual’s ability to deal with and recover from adversity and stress experienced in the past month, including adaptability to change, overcoming challenges, and confidence in managing difficulties. Scores range from 0 to 40, with higher scores indicating greater resilience. Cronbach’s alpha for resilience was α = 0.78.

### Salivary cortisol

In W1, after the assessment session, all the participants were given detailed written instructions to collect cortisol samples at home by themselves. They were asked to collect a total of four saliva samples at home immediately after awakening and 15-, 30-, and 45-min post-awakening on two consecutive weekdays using Salivettes (SARSTEDT, Nümbrecht, Germany). To ensure compliance with the sampling protocol, the cotton from the Salivettes was stored in MEMS TrackCap Containers (MEM 6 TrackCap Monitor, Aardex Ltd., Switzerland) to objectively verify the time of saliva collection. In addition, participants recorded the collection times in a log. However, no objective verification of awakening times was conducted, which represents a deviation from the recommended guidelines of Stalder et al. (2016). Participants were instructed to keep the Salivettes in a refrigerator until they delivered them to the laboratory. Moreover, participants were instructed to drink only water and not eat, smoke, or take any stimulants (such as coffee, cola, caffeine, tea, or chocolate) until they had completed the morning saliva samples from waking until 45 min later.

When the samples arrived at the laboratory, they were kept in the refrigerator until they were centrifuged (4000 rpm for 15 min). After the centrifugation, samples resulted in a clear liquid with low viscosity that would be stored at -80ºC for posterior analyses. These biochemical analyses were carried out in duplicate with the salivary cortisol ELISA kit from Salimetrics (Newmarket, UK). Assay sensitivity was > 0.007 ug/dL. Inter- and intra- assay variation coefficients were all below 8%. Cortisol levels were expressed in nmol/L.

### Statistical analyses

The CAR was calculated as the area under the curve with respect to the increase (AUCi), and the total post-awakening secretion was calculated as the area under the curve with respect to the ground (AUCg) from the 0-, 15-, 30-, and 45-min cortisol samples (see Pruessner et al. 2003 for formulas). In six cases, one saliva sample was missing out of the eight collected. In these cases, since the missing data accounted for less than 20% of the total, the missing values were imputed using the expectation-maximization method. The CAR and AUCg values were missing for two participants who had more than one missing saliva sample, and these participants were excluded from the analyses. In addition, cortisol values were log transformed because they did not follow a normal distribution.

Before performing the statistical analyses, participants who scored ± 3 SD from the mean were identified and winsorized by replacing their values with the largest (or smallest) value without outliers, following the Value Modification Method (Kwak and Kim 2017). Replaced data corresponded to four participants with 2 values on FCSRT TIR total (one outlier in W1 and one in W2), four participants with 5 values on TMT total (one outlier on TMT-A W1; two on TMT-B W1, and two on TMT-B W2), and one participant on the SFT in W1.

Paired *t*-test analyses were performed to assess differences on all the cognitive tests between W1 and W2, in order to view possible changes across time in the cognitive function and resilience in the entire sample.

To investigate the association between post-awakening cortisol levels and cognitive decline, a new variable was computed by subtracting W2 performance from W1 performance (W1-W2) for each cognitive outcome, representing the magnitude of cognitive decline. Partial correlations were then performed to examine whether this decline was associated with the CAR and AUCg. Additionally, sex, age, and awakening time (AT) were included as covariates to control for their potential influence on this relationship. To control for potential non-adherence effects, analyses were conducted twice: first with the total sample (total sample CAR) and then exclusively with the 2-Day CAR group (see Sect. 3.1).

Finally, following Preacher et al. (2007), a mediation analysis was performed to assess whether resilience mediates the relationship between the CAR and the change in semantic fluency, according to the results obtained in the regression analyses. There is some consensus that there should only be one requirement to establish mediation: the indirect effect (a*b) has to be significant (Zhao et al. 2010; Hayes 2017). We entered post-awakening cortisol secretion at W1 as the independent variable, resilience as the mediator, and cognitive decline (W1-W2) in both phonemic fluency and semantic fluency as the dependent variable. Furthermore, we included age, sex, and AT as covariates. The bootstrap data resampling procedure draws random samples of a fixed sample size with replacements from the dataset, which increases the statistical power. This type of statistical approach partially corrects the sample size issue and controls for this factor in the analyses (Hayes 2017).

We used the Hayes PROCESS macro (Hayes 2017), specifically model number 4, with SPSS (version 28; IBM Corporation, Armonk, NY, USA). All *p* values were two-tailed, and the level of significance was taken as *p* < 0.05.

## Results

### Adherence to the salivary sampling protocol

It has been acknowledged that the timing of the first saliva sample impacts the reliability of the CAR measurement, and so strict control is necessary. Previous studies indicate that a delay in collecting the first saliva sample might result in a negative CAR, characterized by a decrease in cortisol levels after awakening (Thorn et al. 2006). Following these authors, we identified the possible non-adherent subjects in the same way as our group did in previous research (Almela et al. 2012; Hidalgo et al. 2016; Puig-Perez et al. 2016; Pulopulos et al. 2016a, b; Zapater-Fajarí et al. 2021). Thus, to control this issue, we divided the sample into two groups: (i) 2-Day CAR group: those who had a positive CAR (i.e., cortisol AUCi > 0) on both days, and (ii) 1- or 0-Day CAR group: those who had a positive CAR on only one day or none. Of the total sample, 46.15% of the participants showed a positive CAR on both days (16 women and 8 men), 36.54% of the participants showed a positive CAR on only one day (8 women and 11 men), and the other 15.38% of the participants did not show a positive CAR on either of the two days (5 women and 3 men). These results were obtained by including all 53 participants because, even after excluding the 3 participants who scored less than 27 on the MMSE, we obtained the same result.

**Fig. 1:**
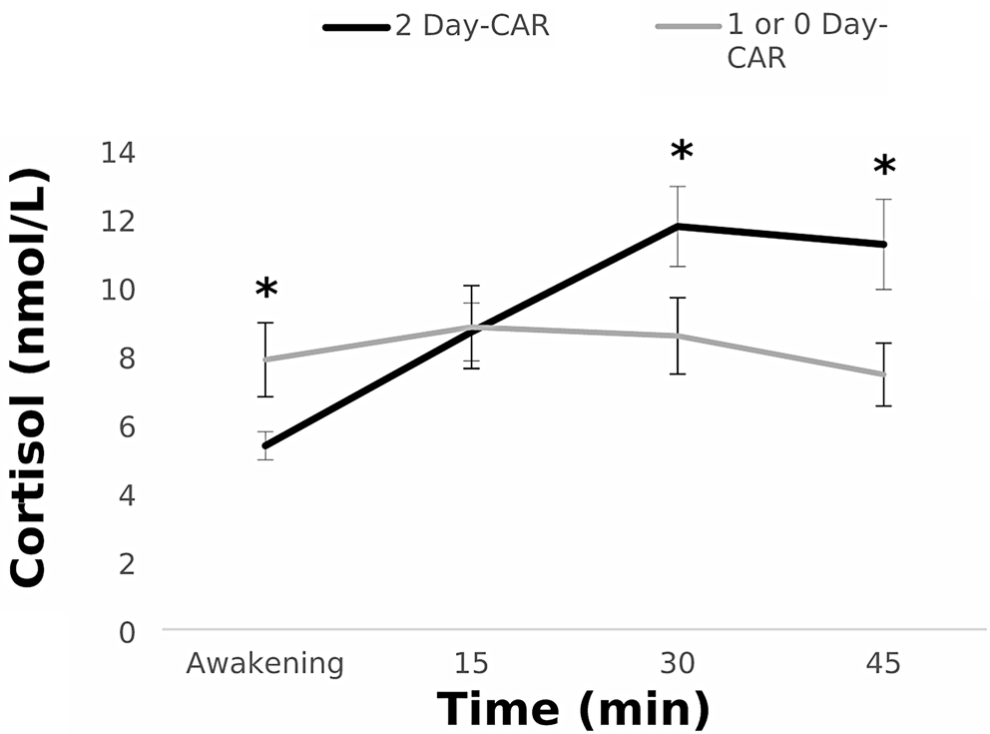
2-Day CAR and 1- or 0-Day CAR groups. Depicted values are means, and error bars represent SEM. *(*p* < 0.05)

To control for a possible effect of non-adherence to the protocol, we repeated the analyses, first with the complete sample (Total sample CAR), and then only with the 2-Day CAR group. This differentiation was made because participants with 1- or 0-Day CAR might have experienced a delay in collecting the first saliva sample, although other explanations, such as unreported diseases, cannot be ruled out. The cortisol profiles of both groups are illustrated in Fig. 1.

### Differences in cognitive function and resilience between W1 and W2

Regarding cognitive function, significant changes were only observed between the waves in verbal fluency, more specifically in semantic fluency (W1: M = 23.33, SD = 6.2; W2: M = 20.84, SD = 6.18; *t*(37) = 2.998, *p* = 0.004), indicating a decline in performance in the four-year period, given that in W2 the performance was worse than in W1. No significant differences were observed in the remaining cognitive measures between the two waves (see Table 1).

**Table 1:** Mean values (M), standard deviations (SD), and dependent *t*-test for resilience and cognitive tests in wave 1(W1) and wave 2 (W2) and the differences between them (W1-W2)

	W1 M (SD)	W2 M (SD)	W1-W2 M (SD)	t	*p*
CD-Risc	29.74(5.34)	28.18(5.64)	1.56 (5.18)	2.130	0.038
FCSRT TIR	44.92(3.43)	44.41(4.18)	0.49 (4.3)	0.861	0.394
PFT	40.73(10.33)	42.63(8.5)	-1.9 (9.6)	-1.415	0.163
SFT	23.33(6.2)	20.84(6.18)	2.51 (5.98)	2.998	0.004
TMT A	39.51(11.58)	41.47(13.8)	-1.96 (12.27)	-1.141	0.259
TMT B	78.26(21.49)	76.80(25.24)	1.45 (24.67)	0.420	0.676
Stroop	5.57(7.7)	7.39(10.26)	-1.83 (10.13)	-1.223	0.228

Note: FCSRT TIR = Total Immediate Recall; PFT = Phonological Fluency Test; SFT = Semantic Fluency Test; TMT = Trail Making Test

In contrast, the scores on the resilience questionnaire were higher in W2 (M = 28.18, SD = 5.64) than in W1 (M = 29.74, SD = 5.34) (*t(49)* = 2.31, *p* = 0.038).

### Relationship between post-awakening cortisol levels and cognitive decline

Results of the partial correlations analyses did not show a predictive role of CAR in any of the cognitive change outcomes (all *p* > 0.12) (See Table 2). However, the total post-awakening cortisol secretion was negatively related to both phonemic and semantic fluency decline (*p* < 0.01 and *p* < 0.04, respectively) (see Table 2).

**Table 2:** Partial correlation analysis between CAR and AUCg and cognitive changes, controlled by sex, age and awakening time for both total sample CAR and 2-Day-CAR groups for CAR index and AUCg only for total sample CAR group

	CAR				AUCg	
	Total sample CAR		2-Day-CAR		Total sample CAR	
	r	*p*	r	*p*	r	*p*
FCSRT TIR Change	-0.09	0.59	-0.35	0.12	-0.30	0.07
PFT Change	-0.19	0.26	-0.22	0.34	-0.46	0.004
SFT Change	0.01	0.94	0.05	0.83	-0.35	0.04
TMT A Change	-0.18	0.29	-0.11	0.64	-0.23	0.16
TMT B Change	-0.11	0.49	-0.35	0.12	-0.05	0.78
Stroop Change	-0.21	0.20	0.23	0.32	-0.20	0.24

Note: FCSRT TIR = Total Immediate Recall; PFT = Phonological Fluency Test; SFT = Semantic Fluency Test; TMT = Trail Making Test

### Testing the mediation model: the role of resilience

Resilience was tested as a mediator in the association between total post-awakening cortisol secretion and phonemic or semantic fluency performance. Resilience only played a mediational role in the relationship between AUCg and semantic fluency decline, but not in phonemic fluency decline. Specifically, mediation analysis showed that higher total post-awakening cortisol secretion was associated with higher resilience (path *a*: B = 4.63, SE = 1.88, *p* = 0.02), and resilience, measured at W1, was significantly and negatively associated with semantic fluency decline (path b: B = -0.33, SE = 0.16, *p* = 0.04), whereas no such association was observed for phonemic fluency decline (see Table 3).

**Table 3:** Mediation model to test the indirect effect of AUCg on decline on the phonemic fluency decline via resilience. **Dependent variable** (Y): phonemic fluency decline. **Mediator (M)**: resilience

	Effect	SE	t	*p*	LLCI	ULCI
a	4.63	1.88	2.47	0.02	0.84	8.43
b	0.3	0.26	1.17	0.25	-0.22	0.82
c’	-11.21	3.24	-3.46	< 0.01	-17.77	-4.66
ab	1.4	1.36			-0.53	4.74

Note: Letters represent the relationship between post-awakening cortisol levels and resilience (a), the relationship between resilience and semantic fluency decline (b), and the direct effect (c’) and the indirect effect (ab) for each condition. SE = standard error; LLCI = lower level of confidence interval. ULCI = upper level of confidence interval

**Fig. 2:**
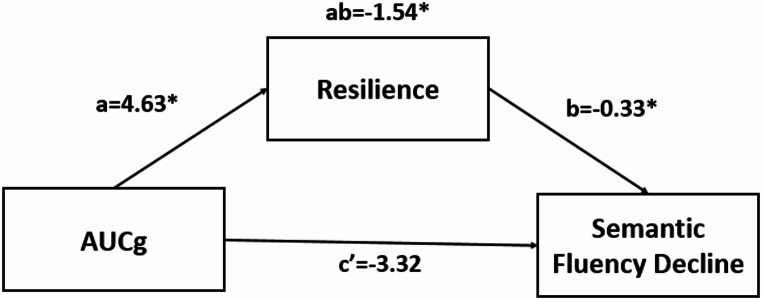
Mediation model to test the indirect effect of total post-awakening cortisol levels on the semantic fluency decline via resilience. **Independent variable (X)**: Total post-awakening cortisol levels. **Dependent variable (Y)**: Semantic Fluency Decline. **Mediator (M)**: Resilience. Note: *p < 0.05

The direct effect, which examines the relationship between total post-awakening cortisol secretion and semantic fluency decline while accounting for resilience, did not show any association (path c’: B = -3.32, SE = 2.01, *p* = 0.11). The conditional indirect effect of total post-awakening cortisol secretion on semantic fluency decline through resilience was significant and negative (path ab: B=-1.54, SE = 0.89, LLCI= -3.6, ULCI=-0.04) (See Table 4; Fig. 2). In contrast, the conditional indirect effect on phonemic fluency decline through resilience was not significant (see Table 3).

**Table 4:** Mediation model to test the indirect effect of AUCg on decline in the semantic fluency decline via resilience. **Dependent variable** (Y): semantic fluency decline. **Mediator (M)**: resilience

	Effect	SE	t	*p*	LLCI	ULCI
a	4.63	1.88	2.47	0.02	0.84	8.43
b	-0.33	0.16	-2.19	0.04	-0.65	-0.01
c’	-3.32	2.01	-1.65	0.11	-7.39	0.76
ab	-1.54	0.89			-3.6	-0.04

Note: Letters represent the relationship between post-awakening cortisol levels and resilience (a), the relationship between resilience and semantic fluency decline (b), and the direct effect (c’) and the indirect effect (ab) for each condition. SE = standard error. LLCI = lower level of confidence interval. ULCI = upper level of confidence interval

## Discussion

The present study was designed to investigate the impact of post-awakening cortisol (specifically CAR and total post-awakening cortisol secretion, AUCg), measured at baseline (W1), on cognitive domain changes during a four-year follow-up period in healthy individuals, particularly in cognitive domains dependent on prefrontal cortex and hippocampus functioning. In addition, we aimed to explore the mediating role of resilience in this association. The main results of our study were: (i) a decline was only found in the semantic fluency domain; (ii) total post-awakening cortisol secretion was negatively associated with both phonemic and semantic fluency decline; and (iii) resilience had a mediating effect on this association; that is, higher total post-awakening cortisol secretion was related to higher resilience, which, in turn, was related to less semantic fluency decline.

To achieve this, we first compared the results from the four-year follow-up study to identify specific domains where participants showed a decline. We explored this decline in cognitive domains dependent on prefrontal cortex and hippocampus functioning. For prefrontal cortex-dependent functions, we examined executive functions according to the paradigm established by Diamond (2013), which delineates three subcategories: inhibition, working memory, and cognitive flexibility. We found some stability in these components, except for a decline in verbal fluency, specifically on the semantic fluency test. According to previous literature, semantic fluency is more strongly associated with age (Kozora and Cullum 1995; Crossley et al. 1997) than phonemic fluency, suggesting that it is more vulnerable to declining with senescence. Following Troyer et al. (1997), optimal verbal fluency performance involves generating words within a subcategory (referred to as clustering) and then shifting to a new subcategory once the current one has been exhausted. Clustering is an automatic process that primarily involves semantic categorization associated with the temporal lobe, including declarative memory and the hippocampus (Troyer et al. 1998a, b). In contrast, switching between subcategories is a more effortful process linked to cognitive flexibility and the prefrontal cortex. This distinction is evident in patients with frontal lobe dysfunction, such as those with Parkinson’s disease (Troyer et al. 1998a, b), Huntington’s disease (Rich et al. 1999), multiple sclerosis (Tröster et al. 1998), and schizophrenia (Robert et al. 1998). Regarding hippocampal-dependent functions, we did not observe an expected decline in episodic memory (i.e., total immediate recall). This lack of a decline may be explained by the age distribution of our sample, which includes individuals both above and below the typical age of 70 when short-term memory decline is expected (Grégoire and Van der Linden 1997). Additionally, the total immediate recall index has been suggested to be more sensitive to memory impairments associated with preclinical AD rather than those found in normal aging, which characterizes our sample (Papp et al. 2015).

Our initial hypothesis posited that higher CAR or, notably, higher total post-awakening cortisol secretion measured at baseline, would correlate negatively with prefrontal cortex-dependent decline (greater maintenance) and positively with hippocampal-dependent decline (a greater decline), based on existing literature suggesting cortisol’s influence on various cognitive domains (Almela et al. 2012; Evans et al. 2012; Moriarty et al. 2014; Geerlings et al. 2015; Hidalgo et al. 2016). However, in our sample, we could only verify cortisol’s association with phonemic and semantic fluency decline. As previously noted, the semantic fluency task involves set-shifting, a component of executive function that is strongly influenced by morning cortisol (CAR in this context) (Evans et al. 2012). Nevertheless, this positive correlation between morning cortisol and cognitive maintenance of semantic fluency was evident only in the total post-awakening cortisol secretion (AUCg) and not in the CAR. This result could be due to the fact that, although they are mathematically related and both exhibit significant heritability (Kupper et al. 2005; Wüst et al., 2000), AUCg is less dynamic and more stable than CAR (Stalder et al. 2016), especially when measured over multiple days (Hellhammer et al. 2007), which may enhance its reliability as a longitudinal predictor. This finding is consistent with Dierolf et al. (2016), who suggested that the relationship between cortisol and set-shifting may be more closely tied to mean morning cortisol levels than to the CAR alone. Nonetheless, some controversy remains, given that Greendale et al. (2000) found that higher cortisol levels were associated with a decline in verbal fluency. However, their sample had a higher average age than ours, with a mean of 71 years, and it included only postmenopausal women. Additionally, the cortisol levels were measured in blood, which could have influenced the results. Despite a strong correlation between free cortisol levels in blood and saliva, approximately 30% of free cortisol in blood is converted to cortisone in saliva, potentially resulting in lower saliva levels (Levine et al. 2007). Regarding hippocampal-dependent functions, we did not find a significant influence of cortisol on these functions in the four-year period. Prior literature had suggested a negative impact of CAR on declarative memory due to potential glucocorticoid effects on the hippocampus (Almela et al. 2012; Hidalgo et al. 2016). Notably, these previous studies were predominantly cross-sectional, whereas our study is longitudinal.

Our second objective was to explore the role of resilience as a mediator in the link between post-awakening cortisol levels and cognitive decline. We hypothesized that resilience would positively mediate this association, particularly preserving cognitive function. Based on our findings, we applied this model specifically to the association between total post-awakening cortisol secretion and the decline in semantic fluency, the domain where AUCg showed a significant predictive relationship. Additionally, we examined its association with changes in phonemic fluency, where AUCg also predicted change, albeit not significantly. Mediation analysis revealed a positive effect of resilience exclusively in the association with semantic fluency decline. Thus, higher total post-awakening cortisol secretion was related to higher resilience and, at the same time, less semantic fluency decline. This finding aligns with previous research by Wolf et al. (2019) and Yang et al. (2021) indicating a positive association between resilience and cognitive performance in both middle-aged and older adults. Moreover, Peeters et al. (2023) emphasized the significance of resilience throughout various stages of cognitive decline, and the WHO (2022) highlighted resilience as a critical public health priority. Resilience buffers against stress (Saez-Sanz et al. 2023), reducing the risk of depression and mortality while enhancing quality of life and self-perception (MacLeod et al. 2016). Additionally, resilience has emerged as a crucial protective factor throughout cognitive aging, mitigating the emotional and physiological impact of stress, especially in the face of persistent or cumulative stressors. Regarding cognitive performance, Fazeli et al. (2019) found better performance on working memory, executive functions, and verbal fluency—domains dependent on the prefrontal cortex—in resilient Human Immunodeficiency Virus-positive individuals of similar ages (mean 51.73 years old). Additionally, Wang et al. (2022) demonstrated a relationship between resilience and working memory in elderly individuals. Semantic fluency, moreover, serves as a critical marker for Alzheimer’s disease (Bastin and Salmon 2014), along with working memory (Kessels et al. 2011). Thus, resilience may serve as a socio-cultural indicator of cognitive reserve, due to its association with working memory (Saez-Sanz et al. 2023), supporting the idea that cognitive reserve acts as a protective factor against dementia and Alzheimer’s disease (Stern et al., 2021). Furthermore, Bonetti et al. (2019) identified both working memory and semantic fluency as being dependent on the prefrontal cortex, a key region in resilience (Eaton et al. 2022).

This study presents strengths and limitations that warrant consideration. It contributes valuable insights that can fill gaps in the scientific literature on the longitudinal relationship between post-awakening cortisol levels and cognitive performance (Ouanes et al. 2020), emphasizing the significance of resilience in this association. Furthermore, rigorous cortisol collection procedures were followed, based on the guidelines by Stalder et al. (2016) and updated recommendations by Stalder et al. (2022, 2024), including sample collection on consecutive workdays to ensure accurate results. Saliva sampling was employed for cortisol extraction, recognized for its reliability in revealing cortisol dynamics (Schulz et al. 1998; Melamed et al. 1999; Powell et al. 2002; De Vente et al. 2003; Grossi et al. 2005; Söderström et al., 2006). However, the study has some limitations, such as a relatively small sample size due to challenges in recruiting participants for follow-up waves, largely attributable to the constraints imposed by the COVID-19 pandemic. Additionally, further studies could have provided valuable insights related to CAR by collecting post-awakening cortisol not only one time (W1), but also a second time (W2). These limitations should be addressed in future research to enhance the robustness and applicability of the results.

In conclusion, our study contributes to understanding the complex interplay between post-awakening cortisol levels, cognitive decline, and resilience in healthy individuals over a four-year period. Our findings suggest that higher post-awakening cortisol levels, specifically total post-awakening cortisol secretion measured at baseline, are associated with less semantic fluency decline, a cognitive domain dependent on the prefrontal cortex, over a period of four years. This positive association underscores cortisol’s potential protective role in maintaining cognitive abilities that depend on the prefrontal cortex. Moreover, our results highlight resilience as a mediator in the relationship between cortisol and cognitive change, particularly in semantic fluency, which is of great concern in some clinical conditions with frontal lobe dysfunction. These findings emphasize the importance of considering psychosocial factors such as resilience in understanding the intricate relationship between cortisol levels and cognitive functioning.

Understanding the role of resilience in cognitive performance and in maintaining cognitive performance, particularly in prefrontal cortex-dependent domains, could provide deeper insights into developing interventions for cognitive impairment and highlight resilience as a protective factor (Saez-Sanz et al. 2023).
